# Supplementary material for: Curated and harmonised transcriptomics datasets of interstitial lung diseases
Source: Data Brief. 2025 Oct 14;63:112139. doi: 10.1016/j.dib.2025.112139 (PMC12581653; doi:10.1016/j.dib.2025.112139)
Supplement: Supplementary file 1 [file mmc1.zip › Supplementary_material/RNA-seq/fastQC reports/GSE180415/TRIMMED_SRR15194703_2_fastqc.html]

TRIMMED\_SRR15194703\_2.fastq.gz FastQC Report 

FastQC Report

Wed 25 Oct 2023  
TRIMMED\_SRR15194703\_2.fastq.gz

## Summary

- Basic Statistics
- Per base sequence quality
- Per sequence quality scores
- Per base sequence content
- Per sequence GC content
- Per base N content
- Sequence Length Distribution
- Sequence Duplication Levels
- Overrepresented sequences
- Adapter Content

## Basic Statistics

| Measure | Value |
| --- | --- |
| Filename | TRIMMED\_SRR15194703\_2.fastq.gz |
| File type | Conventional base calls |
| Encoding | Sanger / Illumina 1.9 |
| Total Sequences | 39373302 |
| Sequences flagged as poor quality | 0 |
| Sequence length | 60-80 |
| %GC | 56 |

## Per base sequence quality

## Per sequence quality scores

## Per base sequence content

## Per sequence GC content

## Per base N content

## Sequence Length Distribution

## Sequence Duplication Levels

## Overrepresented sequences

| Sequence | Count | Percentage | Possible Source |
| --- | --- | --- | --- |
| GGGGGGGGGGGGGGGGGGGGGGGGGGGGGGGGGGGGGGGGGGGGGGGGGG | 84228 | 0.21392160606697402 | No Hit |
| GATCAATCGCCCCCGGGGGTGCCTCCGGGCTCCTCGGGGTGCGCGGCTGG | 78361 | 0.1990206460204938 | No Hit |
| GGTGGCGCGTGCCTGTAGTCCCAGCTACTCGGGAGGCTGAGGCTGGAGGA | 67685 | 0.17190582593250625 | No Hit |
| GCGGTGGCGCGTGCCTGTAGTCCCAGCTACTCGGGAGGCTGAGGCTGGAG | 60805 | 0.15443205652398673 | No Hit |
| GGTGGCGCGTGCCTGTAGTCCCAGCTACTCGGGAGGCTGAGGTGGGAGGA | 58854 | 0.14947692220479755 | No Hit |
| CCGGGCTCCTCGGGGTGCGCGGCTGGGGGTTCCCTCGCAGGGCCCGCCGG | 56440 | 0.14334586415942457 | No Hit |
| GCGGTGGCGCGTGCCTGTAGTCCCAGCTACTCGGGAGGCTGAGGTGGGAG | 52307 | 0.13284890355398693 | No Hit |
| CGGTGGCGCGTGCCTGTAGTCCCAGCTACTCGGGAGGCTGAGGCTGGAGG | 45567 | 0.11573070503459425 | No Hit |
| GTTCTGGGCTGTAGTGCGCTATGCCGATCGGGTGTCCGCACTAAGTTCGG | 44136 | 0.1120962625893048 | No Hit |
| CGGTGGCGCGTGCCTGTAGTCCCAGCTACTCGGGAGGCTGAGGTGGGAGG | 39803 | 0.10109134357082879 | No Hit |

## Adapter Content

Produced by FastQC (version 0.11.7)
